# Imaging biomarkers for clinical applications in neuro-oncology: current status and future perspectives

**DOI:** 10.1186/s40364-023-00476-7

**Published:** 2023-03-29

**Authors:** Fang-Ying Chiu, Yun Yen

**Affiliations:** 1 Center for Cancer Translational Research, Tzu Chi University, Hualien City, 970374 Taiwan; 2 Center for Brain and Neurobiology Research, Tzu Chi University, Hualien City, 970374 Taiwan; 3 Teaching and Research Headquarters for Sustainable Development Goals, Tzu Chi University, Hualien City, 970374 Taiwan; 4 Ph.D. Program for Cancer Biology and Drug Discovery, Taipei Medical University, Taipei City, 110301 Taiwan; 5 Graduate Institute of Cancer Biology and Drug Discovery, College of Medical Science and Technology, Taipei Medical University, Taipei City, 110301 Taiwan; 6 TMU Research Center of Cancer Translational Medicine, Taipei Medical University, Taipei City, 110301 Taiwan; 7 Cancer Center, Taipei Municipal WanFang Hospital, Taipei City, 116081 Taiwan

**Keywords:** Glioma, Molecular imaging, Multiparametric MR images, Precision medicine, Quantitative imaging biomarkers, Theranostics

## Abstract

Biomarker discovery and development are popular for detecting the subtle diseases. However, biomarkers are needed to be validated and approved, and even fewer are ever used clinically. Imaging biomarkers have a crucial role in the treatment of cancer patients because they provide objective information on tumor biology, the tumor's habitat, and the tumor’s signature in the environment. Tumor changes in response to an intervention complement molecular and genomic translational diagnosis as well as quantitative information. Neuro-oncology has become more prominent in diagnostics and targeted therapies. The classification of tumors has been actively updated, and drug discovery, and delivery in nanoimmunotherapies are advancing in the field of target therapy research. It is important that biomarkers and diagnostic implements be developed and used to assess the prognosis or late effects of long-term survivors. An improved realization of cancer biology has transformed its management with an increasing emphasis on a personalized approach in precision medicine. In the first part, we discuss the biomarker categories in relation to the courses of a disease and specific clinical contexts, including that patients and specimens should both directly reflect the target population and intended use. In the second part, we present the CT perfusion approach that provides quantitative and qualitative data that has been successfully applied to the clinical diagnosis, treatment and application. Furthermore, the novel and promising multiparametric MR imageing approach will provide deeper insights regarding the tumor microenvironment in the immune response. Additionally, we briefly remark new tactics based on MRI and PET for converging on imaging biomarkers combined with applications of bioinformatics in artificial intelligence. In the third part, we briefly address new approaches based on theranostics in precision medicine. These sophisticated techniques merge achievable standardizations into an applicatory apparatus for primarily a diagnostic implementation and tracking radioactive drugs to identify and to deliver therapies in an individualized medicine paradigm. In this article, we describe the critical principles for imaging biomarker characterization and discuss the current status of CT, MRI and PET in finiding imaging biomarkers of early disease.

**Supplementary Information:**

The online version contains supplementary material available at 10.1186/s40364-023-00476-7.

## Background

**Fig. 1:**
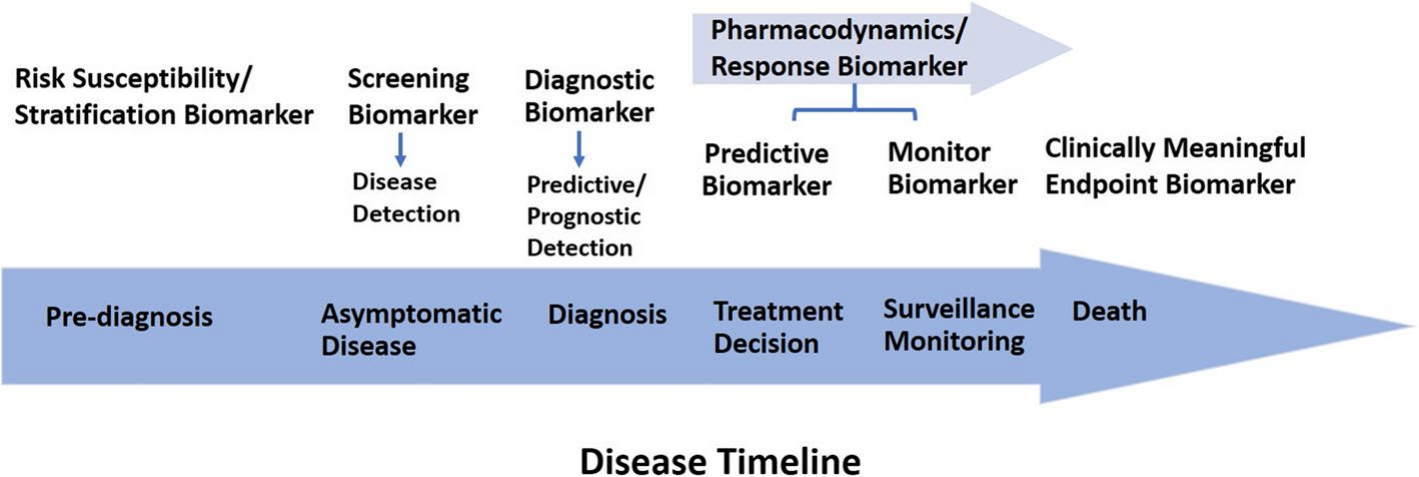
Biomarker categories help to place continuum order in the progression of disease

The 2021 World Health Organization (WHO) classification of tumors of the central nervous system (CNS) included updates with substantial progress in the classification and treatment of gliomas, which will have an impact on treatment and patient monitoring. The changes group tumors into more molecularly and biologically defined entities with better-represented inherent qualities as well as adopting new tumor types and subtypes, especially in pediatric neuro-oncology [1]. The new 2021 WHO classification of CNS tumors has further merged molecular data into the typing, subtyping and grading of major tumor groups. The potency of strengths developed a renovation including more accurate conceptualization of CNS tumor types, improved diagnostic accuracy and reliable prognostic subgroups. As a result, several potential practice-changing clinical trials have recently been completed on the diagnosis and treatment of glioma, and we have entered the epoch of molecular diagnostics, personalized immunotherapy and chemotherapy in precision medicine. According to the CNS 5th edition, the corresponding integration especially affects the classification of adult-type and pediatric-type diffuse gliomas, circumscribed astrocytic gliomas, ependymomas, embryonal tumors and (to a lesser extent) meningiomas [2, 3]. Brain and other nervous system tumors are the leading cause of cancer death among men aged < 40 years and women aged < 20 years [4]. Internationally, early detection and diagnosis are recognized as a crucial priority by a number of organizations, including the WHO and the International Alliance for Cancer Early Detection (ACED). Generally, morphologic changes in tumor size are associated with survival time; they have also been considered as a surrogate endpoint of therapeutic efficacy by the WHO criteria and the response evaluation criteria in solid tumors (RECIST) [5]. The U.S. Food and Drug Administration (FDA) and the National Institutes of Health (NIH), i.e., FDA-NIH Biomarker Working Group, as part of their joint biomarkers, endpoints, and other tools (BEST) resource in 2015, then the next year published the first version of the glossary [6]. The BEST glossary is meant to be a “living” resource that will be periodically updated with additional terms and clarifying information that aims to capture contradistinctions between biomarkers and clinical assessments and to describe their distinct roles in clinical practice, medical product development, biomedical research, and the rules and regulations of products promulgated by the FDA [6–8]. Biomarkers are critical to the practical development of medical diagnostics and therapeutics. Imaging biomarkers have been classified by the NIH initiative on biomarkers and surrogate endpoints as measures that are objectively evaluated as indicators or metrics of normal biological processes, pathogenic processes, or pharmacological responses to a therapeutic intervention. This definition places a major emphasis on the measurements used as markers of a biological state [9, 10]. Several imaging biomarker alliances, such as the European Imaging Biomarkers Alliance (EIBALL) and the Radiological Society of North America (RSNA) Quantitative Imaging Biomarkers Alliance (QIBA®), are setting standards for biomarker development, validation, and implementation, as well as improving the use of quantitative imaging in anatomical, functional and molecular imaging acquisition to obtain image-derived metrics with anatomically and physiologically relevant parameters; likewise, imaging biomarkers will manifest their clinical value in the near future [11–14]. Disease progression and tumor regression identified by imaging biomarkers are significant endpoints. Noninvasive imaging biomarkers, which can diagnose monitor, disease activity, treatment evaluation, decision-making for supporting treatment, and even the clinical endpoint of disease, may have a role in improving this process in routine work. Imaging biomarkers can detect the subtle changes in physiology and pathology in earlier clinical detection and consequently act as surrogate endpoints, reducing the consumption of resources and time in clinical trials [14, 15]. The central role of magnetic resonance imaging (MRI) and positron emission tomography (PET) in neuro-oncology shows their importance in anatomical and metabolic examination. With the focus shifting toward precision medicine, it is of great clinical interest to make accurate quantitative measurements. Substantial advances in imaging analysis techniques, multimodal imaging, the field of quantitative imaging-derived metrics, and imaging biomarker development have shown the maturation of these technologies by demonstrating their clinical value. Particularly, the application of big data analysis by artificial intelligence (AI) is an important branches of computer science and a potential use of digital health care applications in medical imaging [16–19]. In the first part of this article, we discuss the biomarker categories in relation to the courses of a disease and specific clinical contexts (Fig. 1), including illustrating some key MRI and PET biomarkers as well as those currently used in clinical practice for neuro-oncology. The patients and specimens should both directly reflect the target population and intended use of the biomarkers. In the second part, we present the computed tomography (CT) perfusion as a fast and reliable imaging biomarker that sheds light on the physiological role of cerebral hemodynamics in the clinical diagnosis and treatment evaluation of different critical conditions. Additionally, the novel and promising multiparametric MR images approach radiomics features, which are morphological characteristics and brain tumor signatures, will provide deeper insights into how the tumor microenvironment affects biological processes such as immune responses. Furthermore, the diagnostic examination of quantitative data derived from multiparametric imaging, such as diffusion-weighted imaging (DWI), apparent diffusion coefficient (ADC) perfusion-weighted imaging (PWI), and magnetic resonance spectroscopy (MRS) is incorporated into anatomical MRI. In the third part, we briefly address new approaches based on MRI and PET for finding imaging biomarkers combined with applications of bioinformatics, AI, and theranostics in precision medicine. This article covers new research articles and timely reviews on precision-medicine approaches in neuro-oncology.

## Main text

### Biomarker Identification

Biomarkers are indispensable for identifying the mechanisms underlying a disease to find potential new therapeutic crucial targets. They can be used to screen healthy patients for malignancy, estimate the prognosis, predict the outcome from therapy, and monitor disease. In accordance with the National Cancer Institute definition, “a biomarker is a biological molecule found in blood, other body fluids, or tissues that is a sign of a normal or abnormal process, or of a condition or disease”. It may be used to see how well the body responds to a treatment of a the disease or condition, which is called molecular marker or a signature molecule. Moreover, it can be useful in epidemiology to reduce misclassification of exposures and disease, enhance detection of exposure-disease associations, or increase opportunities for intervention, ultimately turning examination findings into a practical application to public health. [20–23]. Identifying and describing biomarkers will help with formulating detailed biomarker descriptions, such as the specific analyte (e.g., fibrinogen), anatomic feature (e.g., joint angle), or physiological characteristic (e.g., blood pressure) that is measured. If applicable (e.g., for molecular biomarkers), the unique identifier of the the biomarker can be described [6, 24]. It has become a powerful tool to classify the risk of progression, identify the severity of the disease, and guide therapy. In addition, biomarkers can be used as indicators of the safety and efficacy of therapeutic interventions or new pharmacological treatments in clinical trials [25]. Several types of biomarker measurements can refer to characteristics such as molecular, physiologic, histologic, and radiographic characteristics. Additionally, these attributes can be objectively estimated and evaluated as an index of normal biological and pathogenic processes or pharmacological responses to a therapeutic intervention. According to the FDA-NIH Biomarker Working Group definition [6], biomarker categories follow alongside the clinical continuum from prediagnosis of disease (prevention), pretreatment (personalized in precision medicine), and even to posttreatment (outcomes and endpoints) as the basis for clinical guidance application [7]. Figure 1 shows a representative summary of biomarker categories placed along this continuum order, and Table 1 provides examples of categories of biomarkers as well as diseases that have been evaluated for thir clinical relevance for various indications.

**Table 1:** Categories of biomarkers and diseases for which different markers have clinical relevance

Types of Biomarkers	Definition	Application	Diseases for Clinical Relevance	References
Diagnostic Biomarker	A biomarker used to detect or confirm presence of a disease or condition of interest, alternatively, to identify individuals with a subtype of the disease	Histology/ Histopathology IDH1/2 mutation 1p/19q codeletion TERT gene promoter mutation MGMT promoter methylation	Oligodendrogliomas: 1p/19q codeletion Glioblastomas: MGMT promoter methylation Gliomas: TERT promoter mutation	[26–31]
Monitoring Biomarker	A biomarker measured continuously for evaluating status of a disease or medical condition or for evidence of exposure to (or effect of) a medical product or an environmental agent	Contrast enhanced MRI brain Circulating exosomes Circulating microRNAs	Brain tumor: MRI Contrast enhanced tumor/nontumor enhancing tissues Brain metastases: Cancer-derived exosomes for Immune interactions and therapeutic implications Chronic lymphocytic leukemia (lymphoid malignancy): MicroRNAs (miRNAs) are a class of small noncoding RNAs	[32–36]
Pharmacodynamic Biomarker / Response Biomarker	Pharmacodynamic and response biomarkers use to reveal that biological response or latently advantageous or harmful, has occurred in an individual who has been exposed to a medical product or an environmental agent As a pharmacodynamic biomarker pharmacologic response in humans, or surrogate endpoint of earlier-phase it depends on characteristic mark by the level of clinical validation	Contrast enhanced MRI brain Reduced malignant cell count in CSF cytology/flow cytometry ^18^F-FDG PET	Brain tumor,metastases in neuroimaging: MRI Contrast enhanced tumor/nontumor enhancing tissues Occult leptomeningeal disease, malignant hematopoietic cells: CNS involvement, cancer cells in the CSF Glioblastoma: Radiopharmaceuticals evaluated in clinical studies for oncology, pharmacodynamic biomarker	[37–49]
Predictive Biomarker	A biomarker used to identify individuals who are more probably than similar individuals without the biomarker to experience an advantageous or disadvantageous effect from exposure to a medical product or an environmental agent	IDH1/2 mutation 1p/19q codeletion MGMT promoter methylation	Glioma: IDH1/2 mutation Oligodendrogliomas: 1p/19q codeletion Glioblastomas: MGMT promoter methylation	[17, 50, 51]
Prognostic biomarker	A biomarker used to identify likelihood of a clinical event, disease recurrence or progression in patients who have the disease or medical condition of interest	IDH1/2 mutation 1p/19q codeletion MGMT promoter methylation SHH gene pathway mutations, Chromosome 17p deletions and TP53 mutations	Glioma: IDH1/2 mutation Oligodendrogliomas: 1p/19q codeletion Glioblastomas: MGMT promoter methylation Medulloblastoma: SHH gene pathway mutations	[52–57]
Susceptibility/Risk Biomarker	A biomarker that reveals the potential for progressing a disease or medical condition in an individual who does not currently have clinically obvious disease or the medical condition	Inherited genetic disorders APOE gene variations DNA repair gene polymorphisms	Alzheimer's disease: APOE genotype	[58, 59]
Safety Biomarker	A biomarker measured before or after an exposure to a medical product or an environmental agent to reveal the likelihood, existence, or range of toxicity as an adverse effect	Complete blood cell count Genetic polymorphisms	Gliomas: Evaluating preoperative complete blood cell count-derived inflammatory biomarkers Diffuse glioma: MGMT gene polymorphisms with myelotoxicity, severe hematological toxicity treated with TMZ	[60, 61]
Validated Surrogate Endpoint	An endpoint supported by an obvious mechanistic rationale and clinical data providing strong evidence that an effect on the surrogate endpoint predicts a specific clinical advantage	Progression-free survival Time-to-progression	Tumor correlate with overall survival in progression-free survival Randomization to tumor progression or death in time-to-progression	[62–64]

These criteria are defined on the basis of the FDA-NIH biomarker working group and contents of a biomarker description [6]

### Diagnostic biomarkers

From the 2016 WHO classification of CNS diseases, the 2021 WHO update makes substantial progress in the classification and treatment of gliomas, incorporating several molecular parameters in addition to the histopathology of formerly and newly defined molecularly defined entities. For instance, isocitrate dehydrogenase (IDH) mutations have different attributes with diagnostic clarity to ensure optimal management and essential regulations for defining clinical trial populations. Assigning the molecular heterogeneity of gliomas and classifying them into dissimilar clinical groups based on codeletion of chromosome arms 1p/19q, IDH mutations, and telomerase reverse transcriptase (TERT) promoter mutations that are characterized by different mechanisms of pathogenesis is important. Previously, glioblastoma (GBM), including both IDH wild-type (90%) and IDH-mutated (10%) tumors, were diagnosed as one entity despite their dissimilar biology and prognosis. Now, diagnostic GBM IDH wild-type diffuse astrocytic tumors in adults only retain one or more of the following three genetic parameters: mutations in the promoter of the TERT, epidermal growth factor receptor (EGFR) gene amplification (tumor-specific aberrations), or both gain of entire chromosome 7 and loss of the entire chromosome 10. Generally, these tumors will be classified as GBM because the of release of circulating tumor cells is associated with EGFR gene amplification, indicating that hematogenous GBM spread out is an intrinsic feature of GBM biology [1, 2, 7, 65]. In addition, the promoter methylation status of the O6-methylguanine-DNA methyltransferase (MGMT) gene has been suggested as the most important predictor of the chemotherapeutic response and patient survival in GBM. Determining MGMT promoter methylation status by multiparametric MRI would help to preoperatively determine the surgical and overall treatment strategy [66, 67]. Diagnostic biomarkers are the key elements to diagnose a disease or a pathogenic process through monitoring situations. It not only serves as a predictive or prognostic marker to support precision medicine but also identifies different subpopulations of patients most likely to benefit from predetermined treatments.

### Monitoring biomarkers

Safety and noninvasiveness are measured continuously or sequentially to detect a changes in the different grades of disease (e.g., imaging). Disease progression and the response of a condition to a treatment, either favorable or unfavorable, the occurrence of new disease effects, can be detected by biomarkers of many categories, e.g., safety biomarkers, pharmacodynamic/response biomarkers, and prognostic biomarkers. These biomarkers would then be used to make treatment decisions and surveil the disease (Fig. 1). MRI examination is a routine work and requisite modality for various studies to monitor disease status, especially before surgery and after treatment in neuro-oncology [68]. The 2015 consensus recommendations for a standardized brain tumor imaging protocol in clinical trials have been developed to help researchers utilize imaging for validation in order to use quantitative imaging surrogates as endpoints in clinical trials for GBM drugs. Moreover, making the best use of different pulse sequences of MR imaging acquisition will further elucidate the tumor's progress and has the potential to improve treatment evaluation with overall survival as the key endpoint [69]. Several studies have shown that circulating tumor cells have been identified in many tumor types and may be perceived in GBM patients. Circulating biomarkers, such as circulating tumor cells, extracellular vesicles and circulating tumor DNA from liquid biopsy are quick, minimally invasive, highly sensitive, and lower-cost samples with the potential non-to help control the treatment of patients with GBM [70]. Tumors shed tumoral content, for instance, circulating tumor cells, cell-free nucleic acids, extracellular vesicles, and proteins, into the circulation, and these biomarkers can cross the blood–brain barrier. Circulating RNAs have been detected in the blood and CSF of glioma patients and may act as biomarkers for diagnosis, prognosis, and treatment monitoring [26, 65, 71, 72].

### Pharmacodynamic biomarkers / Response biomarkers

In recent decades, tumor immunotherapy has shown promise by virtue of its extraordinary efficacy in treating cancer, which has been enabled by the increasing use of molecular imaging. Recently, neoantigen‑based vaccines have demonstrated potential for cancer therapy, primarily by augmenting T‑cell responses. Additionally, targeted radionuclide therapy, namely, molecular radiotherapy, involves a radioactive drug or a radiopharmaceutical that targets cancer cells to assess disease at the molecular level, allowing individual diagnosis [17, 48, 73]. PET imaging can be used in diagnostic settings to identify multimodal molecular and metabolic processes and combines the transport and cellular mechanism of routinely used PET tracers in many neurological diseases, in diagnosis and in the guidance of targeted therapy (Table 2). This molecular imaging modality is used to examine and reveal human physiology through the detection of positron-emitting radiopharmaceuticals. The most popular radiotracers are the short-lived positron-emitting radionuclides ^11^C (half-life, T_1/2_ = 20.4 min), ^13^N (T_1/2_ = 9.97 min), ^15^O (T_1/2_ = 2.04 min), and ^18^F (T_1/2_ = 110 min), with emphasis on the most recent strategies. Principally, ^18^F is most popular to track the high brain uptake of glucose, but its nonspecific uptake in cerebral inflammatory processes hinders its application for diagnosis and brain tumor delineation [74]. Routine PET studies are valuable in diagnostic imaging modalities and in neuro-oncology; likewise, several PET radiotracers and biomimetic materials for drug delivery in theranostics are of foremost concern with biomimetics immoderately taken up by cancer cells in response to elevated proliferation or metabolism [75]. These different types of radiopharmaceuticals, ^18^F-FDG, ^18^F-labeled fluoro-3'-deoxy-3'-L-fluorothymidine (^18^F-FLT), 3,4-dihydroxy-6-(^18^F)fluoro-L-phenylalanine (^18^F-FDOPA), and L-methyl-^11^C-methionine (^11^C-MET), have uptake that is correlated with the clinical indications of neoplasm, solid malignancies, glioma and neuroendocrine tumors, respectively. Radiopharmaceuticals used for hypoxia investigations include ^18^F-labeled fluoromisonidazole (^18^F-FMISO), ^18^F-labeled 2-(2-nitro-1H-imidazol-1-yl)-N-(2,2,3,3,3-pentafluoropropyl)-acetamide (^18^F-EF5), and ^18^F-labeled flortanidazole (^18^F-HX4), which play an important role in solid malignancy pathology and are detected and monitored using PET in clinically relevant conditions. ^18^F-Alfatide II has potential value in detecting brain metastases of different cancers as a biomarker of angiogenesis and is a safety tracer with dosimetry traits. Its advantages are its easy preparation, fast labeling, and in vivo pharmacokinetics. Glycosylated RGD (arginyl-glycyl-aspartic acid) peptide is a tracer for tumor targeting and angiogenesis imaging with improved biokinetics, and integrin α_v_β_3_ plays an important role in tumor-induced angiogenesis and metastasis. This favorable biokinetic configuration establishes glycosylated RGD peptide as a promising lead structure for tracers to quantify α_v_β_3_ expression using PET in neuro-oncology [76]. ^11^C-acetate is generally used in cancers as a biomarker of amyloid-induced neuroinflammation and to evaluate the usefulness of ^11^C-acetate PET in detecting glioma, grading glioma, astrocytoma, meningioma, and monitoring radiosurgery response in meningioma. Radiopharmaceutical choline analogs have been successful as oncological PET probes. In the brain, discrimination between normal tissue and tumors are feasible as a result of the lower physiological uptake of ^11^C-choline or ^18^F-fluoroethyl-choline (^18^F-FCho) by normal brain cells [77, 78]. Gallium-68-labeled FAPI-04 and DOTA-SP have been used as targeting agents in patients with various cancers. For instance, they have been used as small molecule carriers in solid malignant tumors and peptide carriers in glioma. Particularly, ^68^ Ga-labeled fibroblast-activation protein inhibitor (^68^ Ga-FAPI) demonstrated significant uptake in IDH-wildtype GBM and grade III and IV IDH-mutant gliomas [48]. ^89^Zr-PET imaging has led to clinical translation, mostly for antibody or immune-based PET applications. ^89^Zr-bevacizumab and ^89^Zr-fresolimumab are correlated with clinical indications of solid malignancies and gliomas, being antibodies against vascular endothelial growth factor receptor (VEGFR) and transforming growth factor-beta (TGF-β), respectively (Table 2). Tables 1 and 2 were compiled using a systematic approach to collect published studies performed in different models, classified by field of application, specific disease subsection and clinical relevance. Kinetic modeling was able to detect which pharmacodynamic and response biomarkers were safer and faster tracers among the noninvasive biomarkers. In addition, it can identify treatment responders earlier by molecular imaging after treatment initiation. By the same token, it can capture the progression of disease with predictive biomarkers and track the treatment and surveillance of disease with monitoring biomarkers (Fig. 1).

**Table 2:** Radiopharmaceuticals assayed in clinical neuro-oncology studies for PET imaging

Clinical Indication	Radiopharmaceutical	Carrier	Biological Target	References
Neoplasm	^18^F-FDG	Small molecular	Glucose metabolism	[79–81]
Glioblastoma	^18^F-ML-10, ^18^F-ICMT-11	Small molecule	Apoptosis	[82, 83]
Solid malignancies	^18^F-FMISO, ^18^F-EF5, ^18^F-HX4 ^68^ Ga-FAPI-04 ^18^F-FLT ^18^F-Alfatide II ^89^Zr- bevacizumab	Small molecule Small molecule Nucleoside Peptide Antibody	Hypoxia Fibroblast activation protein α Thymidine kinase (DNA replication) Integrin α_v_β_3_ VEGFR	[84–86] [87, 88] [89, 90] [91, 92] [93, 94]
Head and neck cancer	^18^F-PARPi	Small molecule	Poly [ADP-ribose] polymerase 1	[95]
General Cancer / As a Biomarker of Amyloid-Induced Neuroinflammation	^11^C-acetate	Salt	Acetyl-CoA synthetase or Acetate—CoA ^11^C-methionine ligase	[96–98]
Glioma, Neuroendocrine tumors	^18^F-FDOPA, ^11^C-MET or ^11^C-methionine	Amino acid	Amino acid transport	[99–103]
Glioma	^68^ Ga-DOTA-SP ^89^Zr-fresolimumab	Peptide Antibody	Neurokinin 1 receptor TGF-β	[104] [105]

### Predictive biomarkers

To date, in glial tumors, IDH status is the principal molecular feature to evaluate in a glioma, as the absence of IDH mutation worsens the prognosis in both lower-grade gliomas and GBM. Generally, IDH mutation is considered an early marker of gliomagenesis; it leads to overproduction of 2-hydroxyglutarate, which influences cellular metabolism, genetic stability, and epigenetic phenomena in oncometabolites. These essential findings signify that IDH mutation could serve as an important crucial predictive factor for treatment response among glioma patients [106–108]. In the past few years, predictive or prognostic evaluation of glioma has included IDH1/2 mutation, 1p/19q codeletion, and MGMT promoter methylation. IDH mutation and markers have been identified using omics and next-generation sequencing studies. Studies of anaplastic oligodendroglial glioma have shown that 1p/19q codeletion predicts an overall survival advantage from early procarbazine, lomustine and vincristine treatment, which is a combination of chemotherapy drugs for the treatment of glioma [109–111].

### Prognostic biomarker

A prognostic biomarker is measured for a defined biological or clinical attribute to provide information on specific outcome (e.g., disease recurrence, disease progression or overall survival in progression-free survival (PFS), death) independent of treatment received. These biomarkers include IDH1/2 mutations, 1p/19q codeletion status, and MGMT promoter methylation status. In addition, sonic hedgehog gene pathway mutation plays an essential role as a target when there is multifactorial molecular resistance to chemotherapeutic drugs and a poor prognosis and targeted therapies are used against medulloblastoma outcome or disease course in this biomarker [112–114].

### Susceptibility/Risk biomarkers

Susceptibility/risk biomarkers decrease the chance an individual will develop a disease (or medical condition) in the preventive stage. Alzheimer's disease, one kind of dementia, is a complex progressive neurodegenerative disorder characterized by extracellular amyloid β and intraneuronal tau protein aggregations of the brain and is usually detected using diffusion tensor imaging (DTI) MRI, which is a sensitive measure of white matter damage that may predict future dementia risk in cerebral small vessel disease and in mild cognitive impairment. These imaging biomarkers are applied to the preoperative planning for brain tumors, neurodegenerative disease to quantify white matter microstructural changes and ischemic stroke in neuroradiology [115–119]. The apolipoprotein E (APOE) gene is a major risk factor for developing late-onset Alzheimer’s disease, which more frequently appears after the age of 65 years. Humans have three versions of the APOE gene: the ε2, ε3, and ε4 alleles. Carrying the ε4 allele of the APOE gene is the strongest risk factor for late-onset Alzheimer’s disease, while carrying the ε2 allele is protective. APOE-ε4 (APOE4) allele gene polymorphism for early detection is a useful prevention in risk of disease [120].

### Safety biomarkers

These biomarkers give us the ability to detect or predict adverse drug or exposure effects. For example, myelosuppression is the major toxicity encountered during temozolomide (TMZ), a chemoradiotherapy with prognostic significance for GBM. Complete blood cell count is easy and safe to measure to assess myelosuppression in patients receiving chemoradiation or safety to continue administering treatment or not [121]. Genetic polymorphisms in MGMT promoter methylation status have been associated with TMZ-induced myelotoxicity and hematological toxicity in patients with adult diffuse gliomas. Nearly 10% of patients with adult diffuse glioma develop clinically significant myelotoxicity while on TMZ, leading to treatment interruptions. Generally, objective response rate (ORR) and PFS are considered valuable endpoints in clinical trials given their earlier times to events and thus their more proximal relationship to treatment evaluation, prognostic prediction, and impact on clinical decision-making.

### Validated surrogate endpoints

Ideally, a surrogate endpoint fulfills the criterion of a complete relationship between an experimental or therapeutic index and the terminal clinically relevant endpoint of interest. Owing to this relationship at an earlier time point, validated surrogate endpoints may crucially speed therapeutic development for making a strategic decision or patient treatment in clinical works with increasing levels of supportive evidence (Fig. 1). Therefore, the ORR is an important parameter according to WHO and RECIST as an anatomic response criterion based on the visual assessment of tumor size in morphological images provided by MRI or CT, which were developed mainly for cytotoxic chemotherapy in oncology. Recent alternatives to RECIST are immune-specific response criteria for checkpoint inhibitors in metabolic response assessments through PET, which may reflect the viability of cancer cells or functional changes that occur after anticancer treatments [122–124].

### Imaging biomarkers

Currently, medical imaging is a requisite utility in clinical diagnosis and therapy. It includes radiant energy, such as X-rays, computed tomography (CT), and PET, as well as nonionizing radiation, such as MRI. These are sophisticated technologies that utilize a physical mechanism to detect patient internal signals that reflect either anatomical structures or physiological situations. In the past few decades, CT perfusion has been one of the fastest, most common, and valuable imaging techniques applied in clinical diagnosis and evaluation for different conditions. This technique injects an intravenous bolus of contrast agent into the patient, which passes from the arteries through the capillaries to the veins and then into the venous sinuses. Its parameters provide quantitative data such as cerebral blood flow (CBF) calculated from the gradient of the wash-in of the time-pixel density curve and cerebral blood volume (CBV) from the area under the curve, which refers to the volume of blood present at a given moment in a distinct curve area. The mean transit time (MTT) is the time from wash-in to wash-out of contrast agent. MTT is calculated as MTT = CBV/CBF. From the area under the curve, time to peak (TTP) is the time to the apex of the curve and time‐to‐maxima of the tissue residue function (Tmax). Absolute quantification of dynamic contrast-enhanced perfusion parameters can suggest a diagnosis and guide treatment of patients with cerebrovascular conditions, including stroke, vasospasm, moyamoya disease, brain tumors, and traumatic brain injury, in clinical situations to help with clinical decision-making [125–130].

In parallel, advanced imaging techniques are acquiring clinical application, comprising dynamic contrast-enhanced CT perfusion of pharmacodynamic changes and response to diagnosis or treatment evaluation in patients with chronic unilateral high-grade stenosis or occlusion of the carotid or middle cerebral artery [131–134]. CT perfusion has been examined extensively in human stroke studies (e.g., recanalization, reperfusion, and infarct size/growth) as a source of potential imaging biomarkers [135–137]. The regions of the brain with severely reduced CBV or CBF correspond to the regions of core infarction. MTT with prolongation of the perfusion map or its derivatives, the TTP and Tmax of the residue function, have been shown to accurately measure the penumbra in patients with ischemic stroke. The therapeutic time window for acute ischemic stroke has been continually evolving as a surrogate marker for potentially salvageable tissue [138–141]. In addition, imaging-derived properties of benign oligemia, penumbra, and the infarct core of these three hypoperfused tissue compartments have been depicted by mismatch imaging, which can distinguish salvageable tissue invasively by measuring hemodynamics and active metabolism to identify treatable patients using the temporal evolution of CT perfusion metrics within different brain tissue subtypes on neuroimaging. Although DWI is the most accurate technique for infarct detection, CT perfusion still has some strong benefits, such as superior vascular imaging of extracranial and distal intracranial vessels and fewer absolute contraindications.

Available, rapid, and suitable for critically ill patients needing prompt intervention, imaging biomarkers are good for efficacy and safety outcome evaluation, as fast and reliable imaging biomarker interpretations are critical for making influential therapeutic decisions and prognostications in patients with acute ischemic stroke [142, 143]. CT perfusion imaging can provide information about tumor vasculature and in vivo assessment of vascular parameters such as permeability surface area-product (PS) and relative CBV. Conceptually, this noninvasive approach can be evaluated by quantitatively estimating PS, CBV, CBF, and MTT. Several studies have shown that PS and CBV leakiness correlate with histopathologic grade in astroglial brain tumors [129, 144]. As an exception, high-grade gliomas will show higher PS and CBV associations than low-grade gliomas, which can be used to differentiate grade III from grade IV gliomas. Moreover, these measures provide different aspects of tumor microvasculature and tumor biology. Another commonly expressed parameter of vascular leakage is the transfer constant (K^trans^), which depends on plasma blood flow, vascular permeability, and capillary surface area. Since blood flow usually increases very fast in high-grade leaky brain tumors, metabolic demand increases, leading to tissue hypoxia, which in turn induces angiogenesis; hence, K^trans^ approximates PS. Furthermore, PS has been shown to correlate with histological features, microvascular cellular proliferation, and molecular angiogenic markers in gliomas. This is closely related to biological characteristics and consequently may be a surrogate marker for angiogenesis, helping us better understand the role of perfusion imaging as an imaging biomarker [144–146].

Tumor vascularization occurs through several distinct biological processes, representing different tumor types and anatomical locations, and occurs simultaneously within the same cancer tissue. Gliomas are characterized by heterogeneous vasculature relying on angiogenesis to maintain an adequate blood supply and diffuse invasion into the healthy parenchyma. At the cellular level, this microenvironment comprises stem cells, nontransformed/reactive glial (and neural) cells, immune cells, and mesenchymal cells that support tumor growth and invasion through complex network crosstalk. Importantly, glioma angiogenesis and its different hemodynamic features change at the microvasculature plane. CT perfusion in vivo imaging is capable of providing insight into physiological processes, which may provide pivotal supplemental conventional morphologic imaging to predict neuro-oncological outcomes and may also be practical in evaluating the response to various therapies, for a better understanding of tumor biology [129, 144, 147, 148].

Over the past few years, PET/MR has been a promising combination improving PET’s ability to reveal cellular metabolic and molecular circumstances and MRI’s capacity to produce high-resolution images that display organ position, anatomy, function and metabolism in the physiologic state after imaging fusion, all in one image. Particularly, high soft tissue contrast and lower radiation dose appear to be advantages of this novel hybrid modality in the whole-body staging of different cancers and multiparametric tumor imaging. For instance, neuro-imaging has been used to stage brain diseases (i.e., glioma grading, Alzheimer’s disease), and the RECIST criteria were designed to minimize the risk of measurement error and prevent overestimation of response rates of target lesions and their association with overall survival and PFS, as we mentioned earlier. Where MRI is the predominant modality, the lower radiation dose than PET/CT will be particularly valuable in the diagnosis of potentially treatable or curable diseases [149, 150].

**Fig. 2:**
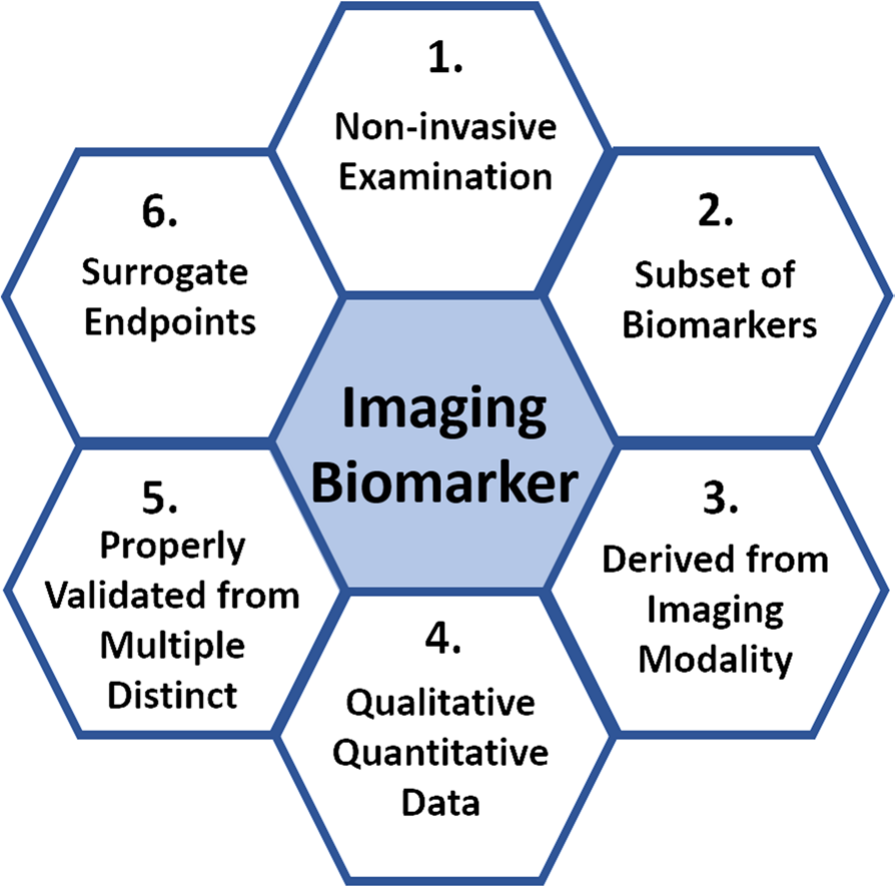
Imaging biomarkers. The key characteristic for the detection, measurement, and application to clinical practice due to the decision-making roles is importantly which is referred to as clinically meaningful endpoint biomarkers derived from different imaging modalities or techniques

As previously stated, categories of biomarkers and diseases for which these dissimilar markers are currently of clinical relevance are listed in Table 1. Molecular-biological probes are important in cancer detection; essentially, carcinogenesis plays a central role in molecular-biological processes that include initiation, promotion, and progression of these three stages when benign neoplasms turn into malignant and invasive lesions. A number of imaging biomarkers are well established in clinical practice. Figure 2 illustrates imaging biomarkers currently used in clinical practice, such as the neurological domains discussed in the utilization of imaging biomarkers that are used for detection, measurement and clinical decision-making, even referred to as clinically meaningful endpoint biomarkers or surrogate endpoints of earlier-phase markers derived from different imaging modalities or techniques. Imaging biomarkers have the key characteristics of noninvasive detection, subsets of biomarkers derived from imaging modalities, and qualitative and/or quantitative data that are properly validated through multiple distinct and surrogate endpoints for detection, prediction, prognostication and response assessment of a disease. MRI (DWI, ADC, PWI, MRS) of quantitative data from extracts in the tumor microenvironment derived from multiparametric imaging and ^18^F-FDG PET absolute values of maximum standardized uptake value at baseline in diagnostic examination. As indicated previously, DTI metrics, i.e., fractional anisotropy (FA), can be employed as surrogate markers for studying cell membrane integrity over time after injury in different models. For instance, ischemic stroke and traumatic brain injury in some cases incite a progressive neuropathology that results in chronic impairments [67, 115, 116, 119, 150, 151].

Moreover, susceptibility-weighted imaging has a variety of applications in neuroradiology practices and routine protocols that can differentiate calcium from brain hemorrhages. Additionally, quantitative susceptibility mapping can be applied to many neurodegenerative disorders by assessing brain iron content, such as diffuse axonal injury (microbleeds), prevenous pathology (multiple sclerosis), dementia (neuritic plaques), high-resolution venous anomaly, and tumor angiogenesis [152–154]. A number of imaging biomarkers are well established in clinical practice for detection (the identification of disease), prediction (the therapeutic outcome/risk of disease), prognostication (the prediction of disease outcome), and response evaluation (the appraisal of variation with therapy). Their evaluation combines molecular features with definitions and the current state of biomarkers in neuro-oncology (Table 1) [15, 155, 156].

### Multiparametric MR images and quantitative information

MR imaging has become one of the requisites for confirmation in a diagnostic approach. It is done by sequential, repetitive data acquisition with fixed parameters. It is able to map anatomy and physiology through noninvasive examinations. In the last few years, MR fingerprinting has become an approved technique for fast, simultaneous quantification of multiple tissue properties in a single, time-efficient acquisition. This technique relies on deliberately varying MR system parameters such that each tissue produces a unique single evolution called a “fingerprint” (i.e., different physical properties that vary from tissue originating in T1-weighted imaging (T1-WI), T2-weighted imaging (T2-WI), proton density and off-resonance frequency). The MR fingerprinting approach makes it possible to glean quantitative information based on digital tissue data and simpler recognition of pathology [157, 158]. The majority of medical images contain vast amounts of information that is encoded in the pixels of digital images and communications in medicine for data storage and transmission of medical images, implementing the integration of medical imaging devices. Multiparametric MR imaging obtains an ideal three-dimensional (3D) image by combining T1-WI, T2-WI, DWI, dynamic contrast enhanced (DCE) and, if necessary, MRS for imaging processing, then moves forward to the image-based biomarker model pipelines for radiomics preparation. These reliable methods integrate anatomical, functional, and metabolic MR imaging as well as may provide a full set of imaging and quantitative biomarker data from several imaging techniques. For instance, using a voxel-based signal intensity method (i.e., the entire volumetric region of T1-WI, T2-WI, DWI, and DCE tumor enhancement) and combining quantitative biomarker data will provide insight into individual tumor habitats and signatures from multiparametric volumetric imaging. Notably, these imaging biomarkers enable qualitative and quantitative traits for biological processes to be addressed on the strength of distinctive image features. Furthermore, comparing patient covariates, liquid biopsy, molecular analysis, and immunohistochemistry with conventional clinical features will facilitate disease diagnosis, treatment evaluation, and prognostic prediction in personalized medicine [159].

### Novel approach in radiomics

In 2010, Robert J. Gillies proposed radiomics as the combination of anatomic imaging and gene expression patterns and depicted the radiology reading room of the future to clearly outline the prospect of radiomics in imaging [160, 161]. Radiomics is an imaging analytical methodology that encompasses the extraction of quantifiable features from big data, which serve as imaging biomarkers for the characterization of tumor phenotypes and pathophysiological processes in the tumor microenvironment that influence their growth, invasion, and metastasis. By extracting and quantifying thousands of imaging features by data mining, which can ascribe characteristics of the cancer phenotype, for example, the tumor's geometrical and morphological characteristics, cluster shade, intensity, diffusion kurtosis, texture, wavelet, etc., to disease entities [162, 163]. In recent years, radiomics has been applied to precision diagnostics and disease stratification or classification in neuro-oncology. It is important to understand the role tumor heterogeneity plays in the natural history of cancer, response to therapy, and even prognostic prediction since primary and metastatic brain tumors are genetic diseases that have histological heterogeneity [164, 165].

### -Omics biomarker identification pipeline for translational medicine

**Fig. 3:**
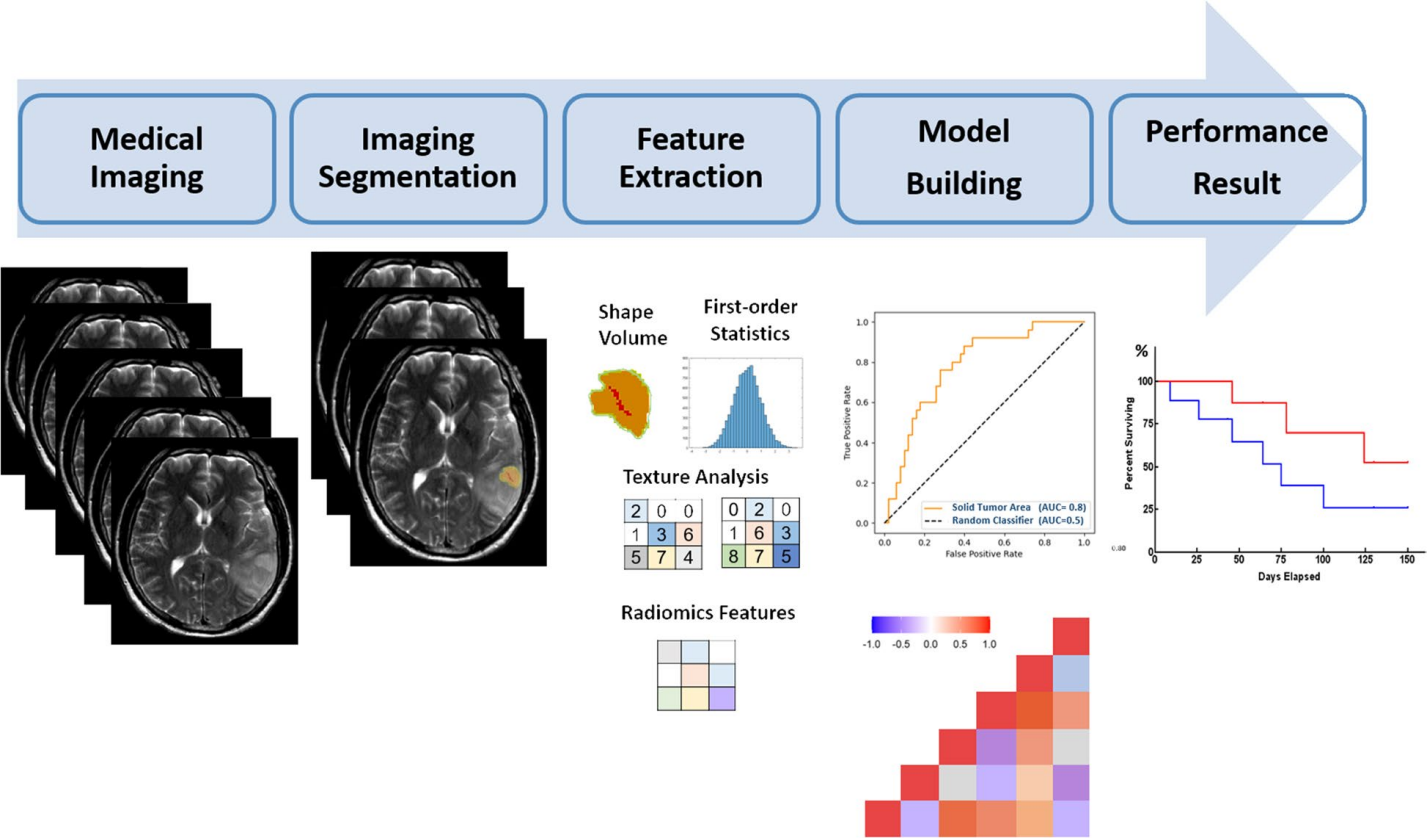
Radiomics workflow. It refers to the extraction of a vast dataset from medical images, such as CT, MRI, and PET images, or synergistic multimodal imaging, which is the process of finding anomalies, patterns and correlations in clinical information to improve diagnosis and predict prognosis with the goal of delivering precision medicine

Radiomics can be performed on images from different modalities, such as CT, MRI, and PET. The images can be manually or automatically processed, followed by image rendering performed on these regions of interest (ROIs). Briefly, an optimal radiomics pipeline involves four steps: (1) imaging acquisition, (2) markup or segmentation, (3) feature extraction, and (4) statistical analysis and classifier model building. The extraction of quantifiable features from big data can identify relevant features for predicting clinical outcomes (Fig. 3). These feature extraction processes can be put into the following categories: (1) first-order statistics: distribution of individual voxel values from an image's histogram of voxel or pixel intensities. (e.g., mean, standard deviation, skewness, kurtosis, energy and entropy). Exceptionally, in non-CNS malignancies, these features have been associated with histological features, tumor grade and subtype [166]. (2) Second-order statistics: statistical interrelationships between neighboring voxels include the so-called textural features to characterize the spatial relationship between voxel intensities, describing the local spatial arrangement of intensities in the image. The most commonly used texture feature is the gray-level co-occurrence matrix, which considers only voxels within a specific range of gray values and makes a matrix of the spatial relationships of pairs of voxels. (3) Shape features: the 3D (or 2D) geometrical composition of the ROI considers the tumor volumetric size from the long axis, solidity, and sphericity to be related to tumor characteristics. (4) Multiscale texture features are higher-order statistics such as the wavelet and Laplacian transforms or Gaussian filtered images. They provide an excellent description of local image variations, such as blobs or edges from rough to subtle texture, and it can also be quantified by parameters such as entropy. Particularly, the wavelet decomposition of an image is based on multiband frequencies to scale the texture inside the image for quantifiers, evaluating the texture of the images as a classifier model [167]. Image-based biomarker model pipelines and radiomics features have become very popular for assessing individual tumor habitats for prognostic significance after chemoradiotherapy and adjuvant therapy for neuro-oncology, such as MRI and PET module identification across complex disease applications in radiology and nuclear medicine, respectively (Fig. 3). More extensively, it is used in medical applications from the diagnosis of micro-level disorders to therapy [168].

### The role of quantitative imaging biomarkers derived from advanced imaging and radiomics in the management of brain tumors

In 2022, Chiu et al. published the first report of multiparametric MR images used to identify and annotate 3D volumetric habitats and signatures in GBM different areas for a machine learning approach [17]. The focus of this article was on the essential role of quantitative imaging in microenvironment habitats, signatures and heterogeneity among tumor subregions in the solid part, peritumoral region, necrosis, and edema. This reflected the regulation of gene expression in necrosis (autophagy genes), peritumoral tissue (vascular endothelial growth factor gene), and edema (angiogenesis genes); in particular, aquaporin-4 water channels contribute to extended tumor cell migration in tumor edema. However, edema was mainly enriched for homeostasis. Collectively, these quantifications of the data demonstrated that the proportional volume size of the edema was approximately 1.5 times larger than the size of the solid part of the tumor; consequently, the volume size of the solid part was approximately 0.7 times that of the necrotic area. It might serve as a potentially quantitative marker for patients with GBM [16, 17]. Comparatively speaking, annotated data are a relatively rare imaging resource and can be costly to acquire. The use of multimodal imaging biomarkers or probes (habitat and signature) can represent the imaging subjects. For instance, diseased tissues unequivocally provide us with more accurate diagnosis and promote therapeutic precision. Particularly, imaging annotation, data extraction, and applying AI systems work in different modes (i.e., learning, reasoning and self-correction skills) for performance, and routine MRI examination may also be significantly associated with anatomical structure, functional physiology, and molecular metabolism of cancer processes and compared with digital pathology, which affects the response to chemotherapy, such as bevacizumab and TMZ treatment (U.S. National Clinical Trials in NIH) in GBM [169].

Similarly, the noninvasive imaging biomarkers may be described as a characteristic feature identifiable on an imaging study that indicates a key disease process from multimodal imaging, such as combining DWI, ADC, PWI, MRS, and PET in the molecular profiling of brain tumors (Table 3). As a practical imaging biomarker (referring to Table 3) considering the pros and cons of distinctive image features in primary research, it holds the potential to enhance robustness in decision support systems. The most aggressive tumors are characterized by angiogenesis. Volumetric measurements of tumor response are better than 2D image measurements of tumor extent, especially for tumors that are irregular in shape, such as GBM. For example, T1-WI provides a high contrast-to-noise ratio between the tumor and surrounding tissue and is pivotal for precise measurement of tumor size. T2-weighted FLAIR sequences (or T2-WI) are recommended for the determination of nonenhancing tumor progression using Response Assessment in Neuro-Oncology (RANO) criteria as a consequence of conspicuous visualization in vasogenic edema, surgery/radiation-induced gliosis, and infiltrating tumors. Remarkably, the anatomical location close to the cortex and ventricles in which CSF can inhibit lesion detection [69].

**Table 3:** Noninvasive clinical imaging biomarkers for detecting evolving entities under the umbrella of theranostics in neuro-oncology

Category in Multilevel Imaging	Imaging Modality / Metric	Distinctive Image Features	Observational Target / Decision-making Role	References
Anatomical structure	CT, MRI: Contrast enhancement	Standard for detection, delineation, and response assessment of tumor High resolution in tumor enhancement, evaluation of the tumor vascularity CT: Ionizing radiation Iodine-based contrast agent (the risk of immediate HSRs) MRI: Non-ionizing radiation Gadolinium-based contrast agent (the risk of NSF)	Identify blood products or calcification within the tumor Prognostic/surrogate imaging biomarker in nearly all cancers	[170–172]
	MRI: T2-FLAIR	To suppress the signal originating from bulk fluid including CSF for better visualization of vasogenic edema Near the cortex and ventricle where CSF can inhibit lesion detection Assessment of white matter tumor involvement and related edema	Detection of subtle changes at the periphery of the hemispheres, periventricular region closes to CSF Best for lesion at brain and CSF interfaces Highly specific imaging biomarker in T2-FLAIR mismatch sign	[173]
Functional physiology	MRI: DWI, DTI, ADC, DKI	Brownian motion of water particles at the microscopic level within biological tissues DTI: isotropy, anisotropy ADC: track the rate of microscopic water diffusion within tissues DKI: dimensionless metric that quantifies the degree of deviation from Gaussian diffusion behavior	Provided neurosurgeons with new tools to overcome the challenge of differentiating healthy tissue from tumor-infiltrated tissue Resective surgery: removing patients with tumors found to be embedded in or in close proximity to functionally important regions; ADC change in response to extracellular (vasogenic; normal or increased diffusion) and cellular (cytotoxic; restricted diffusion) forms of edema Describe the degree of structure of a biological tissue / Enable quantification of non-random diffusion of water due to structural features present in tissues Predictive imaging biomarker before neurosurgery	[174–179]
	MRI: PWI, ASL,DCE, DSC,	Insight into the hemodynamic change by bolus-tracking through the first pass of contrast agent,i.e., either endogenous (arterial water) or exogenous (gadolinium) contrast agents Assessment of blood flow into the mass / tumor to study the brain microvascular component ASL: magnetically labeled the protons in the arterial blood as an endogenous tracer by radiofrequency pulses (without the exogenous injection of contrast media) in technique, i.e., continuous ASL and pulsed ASL, it the most commonly derived is cerebral blood flow information DCE: providing insight into the nature of the bulk tissue properties at the microvascular level compartment (plasma space and extra-vascular-extracellular space) in pharmacokinetic model DSC: performing with intravenous bolus injection of a paramagnetic contrast agent or a particulate agent of superparamagnetic iron oxide using T2-WI or T2*-WI, and the most commonly used MR perfusion technique of the neuro-oncology	Provide clinically useful physiologic information using pharmacokinetic models to complement conventional contrast-enhanced MR imaging, particularly of brain tumors and stroke Diagnosis of tumors and the evaluation of the treatment response in differentiating recurrent tumoral disease from therapy-related necrosis Predictive/progression /prognostic evaluation imaging biomarker with overall survival in progression-free survival, and pharmacodynamic biomarker	[180–184]
Molecular metabolism	MRI: MRS	The most using the proton ( ^1^ H) nucleus because of its high sensitivity and ease of implementation for MR examination Spatial localization techniques (i.e., single-voxel: PRESS, STEAM sequences and multivoxel: chemical shift imaging for phase-encoding) record spectra from regions via spatial distribution of metabolites within the brain	Metabolic markers provide neuroimaging biomarker of normal biological and pathological processes or response to a therapeutic intervention metabolism on biochemical changes, define different metabolic tumor phenotypes MRS in tumor classification, tumors versus nonneoplastic lesions, prediction of survival, treatment planning, monitoring of therapy, and post-therapy evaluation Diagnosis/progression /prognostic evaluation imaging biomarker	[185, 186]
	PET: ^18^F-FDG	PET: optimize/minimize injected dose of radiotracer ^18^F-FDG (T_1/2_ = 110 min) is commonly used to detect metabolically active malignant tumor/lesion (i.e., increased glucose uptake and glycolysis of cancer cells) Gamma rays are emitted and detected by gamma cameras to form a three-dimensional image PET scanners can incorporate a CT or MRI scanner (i.e., PET/CT or PET/MRI) hybrid imaging Depicting metabolic abnormalities before morphological alterations occur and identifying the cellular level changes even the immune cell tracking	Accurate diagnosis and staging are essential for the optimal management of cancer patients Monitoring response to therapy and permitting timely modification of therapeutic regimens Diagnosis/progression /prognostic evaluation imaging biomarker	[187–189]

DWI is sensitive enough for early detection of microscopic, subvoxel water motion, e.g., ischemic injury, infection, and abscess formation. In brain tumors, ADC has been shown to be a surrogate for cellularity in certain conditions. For instance, ADC is inversely correlated with tumor cell density, and DWI measures of ADC may be a useful biomarker for quantifying treatment response. As for its application, it results in relatively restricted diffusion in areas of the tumor by virtue of tightly packed tumor cells. Measures of the ADC can be estimated from DWI data, reflecting the restricted cellularity of water motion. Typically, imaging biomarkers of the response to oncological treatments show an increase in ADC value in the extracellular space or membrane permeability, allowing greater water mobility [190]. Glioma biology has noticeable features such as cellular invasion, cellular proliferation, heterogeneous angiogenesis, and apoptosis. In the general vicinity of an active GBM tumor, the new vasculature is structurally abnormal, resulting in contrast agent leakage from the vascular to the extravascular, extracellular space and increased manifestation of lesions on imaging. Assessment of blood flow into the mass or tumor to study the brain microvascular component is important to understand the physiological changes. DCE imaging has shown the quantitative permeability parameter (vascular permeability and/or blood flow) of volume transfer constant, microvascular permeability reflux constant, distributed volume per unit tissue volume of the tumor entity, and peritumoral edema in different glioma grades, which may assist with the choice of clinical treatment for gliomas, such as the fact that peritumoral edema provides information on the invasion degree of tumor cells [191].

According to the RANO criteria, evaluating the first-line treatment of neuroimaging sequences is critical for imaging biomarker assessment, either in clinical protocols or as part of standard practice for glioma treatment response evaluation in imaging flexibility of adjustment of subsequent imaging techniques. Recent advances in crosslinked radiomics or radiogenomics, such as the tumor microenvironment (e.g., tumor cells will be eliminated by the immune system or will escape detection), provide new insights for future investigation and clinical therapeutics in neuro-oncology. Furthermore, multimodal imaging is associated with individual genomic phenotypes, and a prediction model is constructed through deep or machine learning to provide a paradigm shift toward perspective precision medicine to stratify patients, guide therapeutic strategies, evaluate clinical outcomes, and make predictions from a personalized medicine perspective [192, 193].

As stated above, identifying radiomic or radiogenomic biomarkers provides a platform to investigate tumor heterogeneity by mapping individual tumor habitats. The molecular heterogeneity of gene expression profiles of brain tumors is the most important prognostic factor for drug resistance as well as recurrence. For the above reasons, it is imperative to validate possible target genes related to drug resistance and tumor recurrence, as they can impact the GBM prognostic prediction after chemoradiotherapy and adjuvant courses of TMZ, in order to give the chosen therapy greater prognostic significance in precision medicine, which is the next major challenge that may provide new insights for the treatment of GBM.

### Development of imaging biomarkers and generation of big data

Over the past decade, the role of digital imaging and communications in medicine has been become very important for multimodal imaging with new hardware, sophisticated techniques, and novel protocols. The science and technology of medical imaging are advancing, which has made the most of interchangeable information and achieved the standardization of primarily diagnostic implementation to give imaging a more central role in the context of individualized medicine. Traditional cancer examination takes time and can be painful to the patient. Examples are physical examinations, blood tests, puncture biopsies to stage malignant tumors in differential diagnoses, complete blood cell counts, peripheral blood smears, immunohistochemistry, or even cytogenetic studies. There are now more opportunities to perform multifunctional imaging at a variety of institution types with relatively short examination times. Each technique yields quantitative parameters that reflect specific aspects of the underlying tumor or tissue biology. Many biomarkers have emerged that provide unique information on tumor behavior, including response to treatment. By combining quantitative biomarker data from a number of imaging techniques, one may begin to understand how novel therapies affect tumor cells and tissue microenvironments and be useful for pharmaceutical drug development and therapeutic efficacy prediction. Quantitative imaging modalities (e.g., MRI and PET) are less strongly affected by the scanner, the sequence, and the protocol variabilities. Multiparametric MRI and/or PET imaging, along with the various procedures, generate vast amounts of data for feature extraction, and it will be a herculean task to interpret images in different modalities and compare them with digital pathology. This goes beyond what can be achieved using any single functional technique, thus allowing an improved understanding of biologic processes and of responses to therapeutic interventions [194, 195].

AI applications in bioinformatics have sprung up in all areas of multidisciplinary research in recent years. AI tools radically improve the transparency and robustness of data causal inferences, explanatory power, visualization models, and medical imaging. Deep learning, a subset of machine learning, has augmented capture as a methodology, which has seen effective utilization in decoding image-based perplexities, including those in medical imaging. These contributions from AI methodological interpretation, a documented way of selecting model hyperparameters without ever using data or an unannotated dataset from the test. In addition, multi-institutional data include all patient demographics, disease state and different cohorts or ethnicities, with explicit inclusion criteria for federated learning facilitating multi-institutional collaborations without sharing any identifying data. Ideally, there should be enough data in the test set to give comparisons statistical power, at least several hundred samples, or even thousands of data for the best statistical power. Furthermore, confidence intervals of the reported performance metrics can help establish external validity [196–198]. Different quantitative metrics (e.g., radiomics or radiogenomics) can be used to capture the different viewpoints of the clinical issue, and they can be related to applicable clinical performance metrics to estimate the potential health benefits. By combining quantitative biomarker data from a number of imaging techniques, one may begin to understand how novel therapies affect tumor cells and tissue microenvironments to come nearer to targeted therapy and drug discovery through imaging biomarkers and precision medicine.

### Groundbreaking preclinical imaging in theranostics applications

**Fig. 4:**
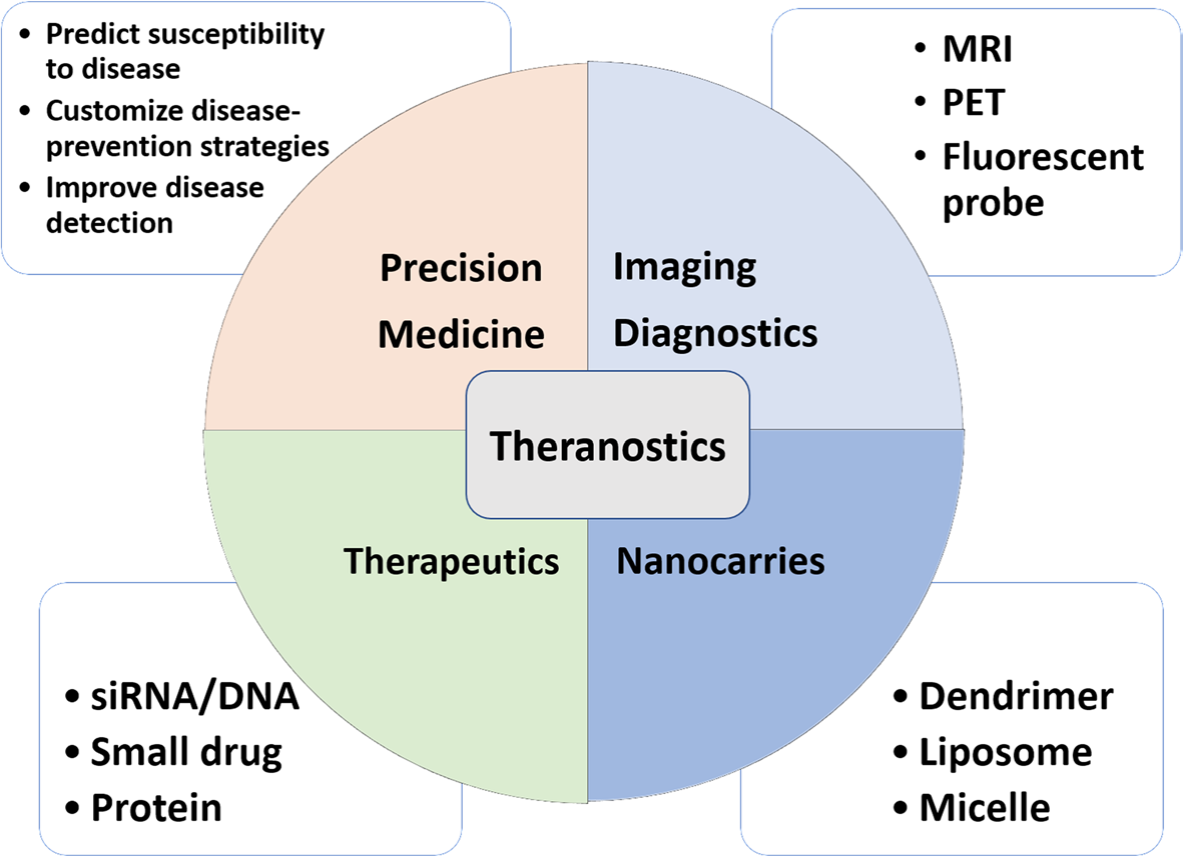
Theranostics. Schematic representation of imaging diagnostics, nanocarriers and therapeutics enlighten future scenario while enabling tailored designs for precision medicine

The European Society of Radiology disclosed its first white paper on personalized medicine, which concluded that medical imaging plays a critical role in several aspects of personalized medicine and revealed that therapeutic applications of imaging within personalized medicine can be categorized into four areas: drug discovery, theranostics, image-guided interventions, drug delivery and therapy monitoring [199]. Surrogate markers and biomarkers in medicine based on imaging readouts that provide predictive information on prompt results are increasingly being used. MRI-derived surrogate assessments are widely used to discriminate gliomas, which represent 80% of all primary malignant brain tumors, including their morphological resemblance to glial cells (such as astrocytes, ependymal cells, microglial cells and oligodendrocytes) as well as grading their malignancy (WHO grade I–IV, from low to high) [200]. Recently, translational bioinformatics methods for drug discovery and drug repurposing (also known as drug repositioning) have shown promise in revealing new prognostic markers for an existing drug in target therapy. Theranostics is a practical concept that involves the integration of diagnosis and therapy in a single platform using nanomaterials for pretargeting drug delivery and imaging devices in precision cancer medicine. Sophisticated techniques merge achievable standardizations into an applicatory apparatus for primarily diagnostic implementation and tracking radioactive drugs to identify (diagnose) and deliver therapies (cure) in the physiological condition of individualized medicine. Theranostics combines MRI-based imaging with high-resolution anatomical structure imaging, the extent of an abnormality, and PET with unlimited penetration depth, high sensitivity in novel targeted immune therapies for hybrid imaging techniques in an interdisciplinary field, aiming toward more controllable, specific, and efficient application [201]. This in vivo approach includes the following:

- (1) Imaging diagnostics, e.g., MRI, PET, and fluorescent probe modalities. Image-guided techniques play a leading role in the study of the quantitative biodistribution and pharmacokinetics of therapeutics or drug delivery systems in a noninvasive method that can provide for dose optimization and treatment monitoring in the development of theranostics. Imaging surrogate assessments with MRI are widely used to characterize gliomas; for example, radiomic analysis offers volumetric features that are significantly associated with various sets of tumor phenotype features and biological processes for prognostication. The radiomics-driven signature serves not only as a predictive biomarker but also as a potential guideline for targeted therapy [17]. The nuclear theranostics approach includes the diagnostic and therapeutic components and their respective radioisotopes with all their advantages; for example, specific agents may be labeled with a γ-emitting radionuclide for PET or single-photon-emission computerized tomography (SPECT) imaging diagnosis, such as the pivotal role of ^18^F-FDG (T_1/2_ = 110 min) and technetium-99 m (^99m^Tc, T_1/2_ = 6 h). Otherwise, combination with an α- or β-particle–emitting radionuclide in radium-223 (^223^ Ra, T_1/2_ = 11.43 days) or yttrium-90 (^90^Y, T_1/2_ = 2.67 days) is suitable for therapy, respectively. In addition, anatomical imaging modalities MRI and molecular imaging PET strategic alliance as well as optical probe imaging fluorescent probe labeled nanoparticle delivery and therapeutic efficacy systems in the newly developing theranostics fields include diagnosis, treatment process monitoring, and tracking the drug delivery either small-molecule drugs or monoclonal antibodies using highly sensitive imaging modality in precision targeted therapy [202, 203].
- (2) Nanocarriers, e.g., dendrimers, liposomes, and micelles. In the last few years, the application of nanoparticles has extended into an expansive variety of tumor-targeted drug delivery based on tumor specific, receptor-mediated endocytosis and phagocytosis. The basic principle of using nanocarrier materials is to incorporate diagnostic and therapeutic entities within a single formation, and image-guided detection has been largely increased, with drug targeting being a valid tool to guide nanoimmunotherapy. In addition to liposomes, polymers have been extensively used for drug delivery purposes for theranostics. For example, by navigating biological barriers in systemic circulation, antibody-targeted nanoparticles reach cancer cells with complementary receptors to navigate the tumor microenvironment and cellular levels, especially tumor-associated macrophages, which are associated with drug resistance and poor prognosis because the tumor microenvironment plays essential roles in immune escape as well as therapeutic failure; these are heterogeneous stratifications based on biomarkers and genetic information of precision medicines [204–206]. Additionally, drug delivery strategies for therapeutic tumors, noninvasive monitoring of their biodistribution, drug release, target-site accumulation, and off-target localization warrant further study. Theranostic nanomedicines carry immense capacity for improving and personalizing anticancer therapy.
- (3) Therapeutics, e.g., small interfering RNA (siRNA)/DNA, small-molecule drugs, and protein applications. The physiological mechanisms of the blood‒brain barrier (BBB) neurovascular unit make up a distinctive and tightly regulated interface between the neuronal environment of the CNS and the peripheral circulation for the preservation of normal brain activity and a stable homeostatic environment. Glioma biology is a noticeable feature of cellular invasion, cellular proliferation, heterogeneous angiogenesis, and apoptosis. Interestingly, GBM characterizes the central figure as endothelial proliferation resulting in tortuous, disordered, and highly permeable vasculature. The tumor microenvironment is essential for immune escape by tumor cells, which plays critical roles in tumor evolution and metastasis. As mentioned above, nanocarriers have been devised to advance the antitumor activity of compounds. One of the essential requisites for intravenously administered nanocarriers for drug delivery could exploit this phenomenon in GBM [207]. Chemoradiotherapy, the concurrent administration of chemotherapy (anticancer drugs) and radiation therapy, is a treatment paradigm in oncology. It is part of the standard of curative and care treatment of various cancers. Several remedies have been designed to improve the antitumor activity of the aforementioned syntheses. siRNA, which stands for either silencing or short interfering RNA, is a molecule that inhibits gene expression and its functions through the RNA interference pathway. Delivery of multiple therapeutic agents and codelivery strategies with siRNA as a potent gene-editing tool in cancer therapy has been shown to have a highly effective antitumor effect. The combination of chemotherapy with RNA schemes to suppress the expression of proteins implicated in the emergence of drug resistance shows promise as a synergistic strategy to reverse or circumvent acquired chemoresistance. Additionally, the combination of natural compounds, gene therapy (siRNA or DNA), small drugs, and proteins is favorable over monotherapy for cancer therapy. In addition, the DNA synthesis pathway can sensitize intrinsically resistant tumors to chemotherapy, namely, reduce the frequency of relapsed tumors. For instance, how to safely and efficiently deliver siRNAs to desired cells and tissues as well as to enhance the performance of siRNAs are related to their stability, activity, specificity, and potential off-target effects. Given its significance, one of the principal targets of cancer research has been to identify agents and various delivery strategies that can improve the superiority of the therapeutic index on chemoradiation outcomes. Likewise, siRNA delivery platforms have been appraised preclinically and clinically, such as dendrimers, peptides, polymers and diverse lipidoids or lipids, for the development of siRNA targeted therapy by research institutes and biotech companies. Additionally, tissues targeted by siRNA and microRNA (miRNA) therapeutics are currently being investigated at the clinical stage [208, 209].
- (4) Precision medicine, e.g., predicting susceptibility to disease, customizing disease prevention strategies, and improving disease detection in translational bioinformatics medicine applications. The future of molecular imaging is one of cutting-edge solutions in personalized medicine. Recently, there have been great advances in polysaccharide matrix-based, controlled therapeutic delivery and target site imaging theranostics for drug delivery combined with medical imaging modalities such as MRI, PET, and fluorescent probes. This is an extremely versatile new type of drug delivery system with targeted tracer development and intuitive applications that make it easier for theranostics to streamline diagnoses and therapies [210]. Molecular imaging is foremost and is essential in theranostics. It reflects the combination of a therapeutic marker with a diagnostic implementation to enable therapy and imaging visualization simultaneously or sequentially. Several advantages in the clinical applications for noninvasive and therapeutic responses over time are designed and built to generate insightful biomarkers with the sensitivity to capture each patient’s unique clinical picture. This niche may offer a promising new approach toward imaging biomarkers in neuroimaging as well as expanding the range of theranostics applications for patient diagnosis, therapy, and personalized management of underlying tumor biology, guiding treatment and supporting personalized refinement. This may offer a favorable new access toward imaging cancer-type specific biomarkers and molecular imaging with the goal of phenotypic theranostics in the future of precision medicine (Fig. 4) [211–215].

## Conclusions

Imaging biomarkers consist of key biologic characteristics found through noninvasive examination. They are a subset of biomarkers derived from imaging modalities, which can be qualitative and/or quantitative, that are properly validated to have the capability, from multiple distinct and surrogate endpoints, to aid in clinical detection, prediction, prognostication, and response assessment at various stages of the disease pathway. Translational medicine, clinical research, and various omics-based fields are employed for biomarker identification. This article links the main studies of imaging biomarkers with anatomy, physiology, and molecular metabolism stratifications with disease diagnosis, pretreatment evaluation, and prognostic prediction under the umbrella of precision medicine for the first time, and it illustrates the potential role of radiomics in biomarker identification as well as a feedback-interacting AI models evaluating quantitative features of medical images and genetic mutations in neuro-oncology applications. Several extractable imaging biomarkers have the potential to support clinical decision-making. After all, it must be recognized that biomarkers can at least help to establish a continuum of order in the progression of a disease. They can assist in diagnosis, can guide treatment decisions, and can be evaluated in clinical trials to validate their capabilities and eventually bring them to routine clinical practice.

## Supplementary Information

**Additional file 1: Fig. S1. **PRISMA flow diagram of the systematic search.

## Data Availability

Not applicable.

## Abbreviations

Acetyl-CoA: Acetyl coenzyme A
ACED: Alliance for cancer early detection
ADC: Apparent diffusion coefficient
AI: Artificial intelligence
APOE: Apolipoprotein E
ASL: Arterial spin labeling
BEST: Biomarkers, EndpointS, and other Tools
^11^C-MET: L-methyl-^11^C-methionine
CNS: Central nervous system
CT: Computed tomography
CSF: Cerebrospinal fluid
DCE: Dynamic contrast enhanced
DKI: Diffusion kurtosis imaging
DTI: Diffusion tensor imaging
DNA: Deoxyribonucleic acid
DSC: Dynamic susceptibility contrast
DWI: Diffusion weighted imaging
EGFR: Epidermal growth factor receptor
EIBALL: European imaging biomarkers alliance
^18^F-EF5: ^18^F-labeled 2-(2-nitro-1H-imidazol-1-yl)-N-(2,2,3,3,3-pentafluoropropyl)-acetamide
^18^F-FCho: ^18^F-fluoroethyl-choline
^18^F-FDG: ^1^^8^F-labeled fluorodeoxyglucose
^18^F-FDOPA: 3,4-Dihydroxy-6-(^18^F) fluoro-L-phenylalanine
^18^F-FLT: ^18^F-labeled fluoro-3'-deoxy-3'-L:-fluorothymidine
^18^F-FMISO: ^18^F-labeled fluoromisonidazole
^18^F-HX4: ^18^F-labeled flortanidazole
^18^F-ICMT-11: ^18^F- labeled (S)-1-((1-(2-fluoroethyl)-1H-[1–3]-triazol-4-yl)methyl)-5-(2(2,4-difluorophenoxymethyl)-pyrrolidine-1-sulfonyl)isatin
^18^F-ML-10: ^18^F-labeled 2-(5-fluoropentyl)-2-methyl malonic acid
^18^F-PARP: ^18^F-labeled Poly[ADP-ribose] polymerase
^18^F-PARPi: ^18^F-labeled PARP inhibitor
FDA: Food and drug administration
^68^ Ga-FAPI: ^68^ Ga-labeled fibroblast-activation protein inhibitor
^68^ Ga-DOTA-SP: ^68^ Ga-labeled dodecane tetraacetic acid-Substance P (SP)
GBM: Glioblastoma
HIV: Human immunodeficiency virus
HSRs: Hypersensitivity reactions
IDH: Isocitrate dehydrogenase
MGMT: O6-methylguanine-DNA methyltransferase
MRI: Magnetic resonance imaging
MRS: Magnetic resonance spectroscopy
miRNA: MicroRNA
NIH: National institutes of health
NSF: Nephrogenic systemic fibrosis
17 p: Short arm (p) of chromosome 17
ORR: Objective response rate
PET: Positron emission tomography
PFS: Progression-free survival
PRESS: Point resolved spectroscopy
PS: Permeability surface area-product
PWI: Perfusion weighted imaging
QIBA: Quantitative imaging biomarkers alliance
RANO: Response assessment in neuro-Oncology
RNA: Ribonucleic acid
RECIST: Response evaluation criteria in solid tumors
ROI(s): Regions of interest(s)
siRNA: Small interfering RNA
SHH: Sonic hedgehog
STEAM: Stimulated echo acquisition mode
T_1/2_: Half-life
T1-WI: T1-weighted imaging
T2-WI: T2-weighted imaging
T2-FLAIR: T2-weighted fluid attenuated inversion recovery
TERT: Telomerase reverse transcriptase
TGF-β: Transforming growth factor-beta
TMZ: Temozolomide
TP53: Tumor protein p53
K^trans^: Transfer constant (K^trans^)
VEGFR: Vascular endothelial growth factor receptor
WHO: World health organization
