# Supplementary material for: Imaging biomarkers for clinical applications in neuro-oncology: current status and future perspectives
Source: Biomark Res. 2023 Mar 29;11:35. doi: 10.1186/s40364-023-00476-7 (PMC10053808; doi:10.1186/s40364-023-00476-7)
Supplement: Supplementary file 1 — Additional file 1: Fig. S1. PRISMA flow diagram of the systematic search. [file 40364_2023_476_MOESM1_ESM.pdf]

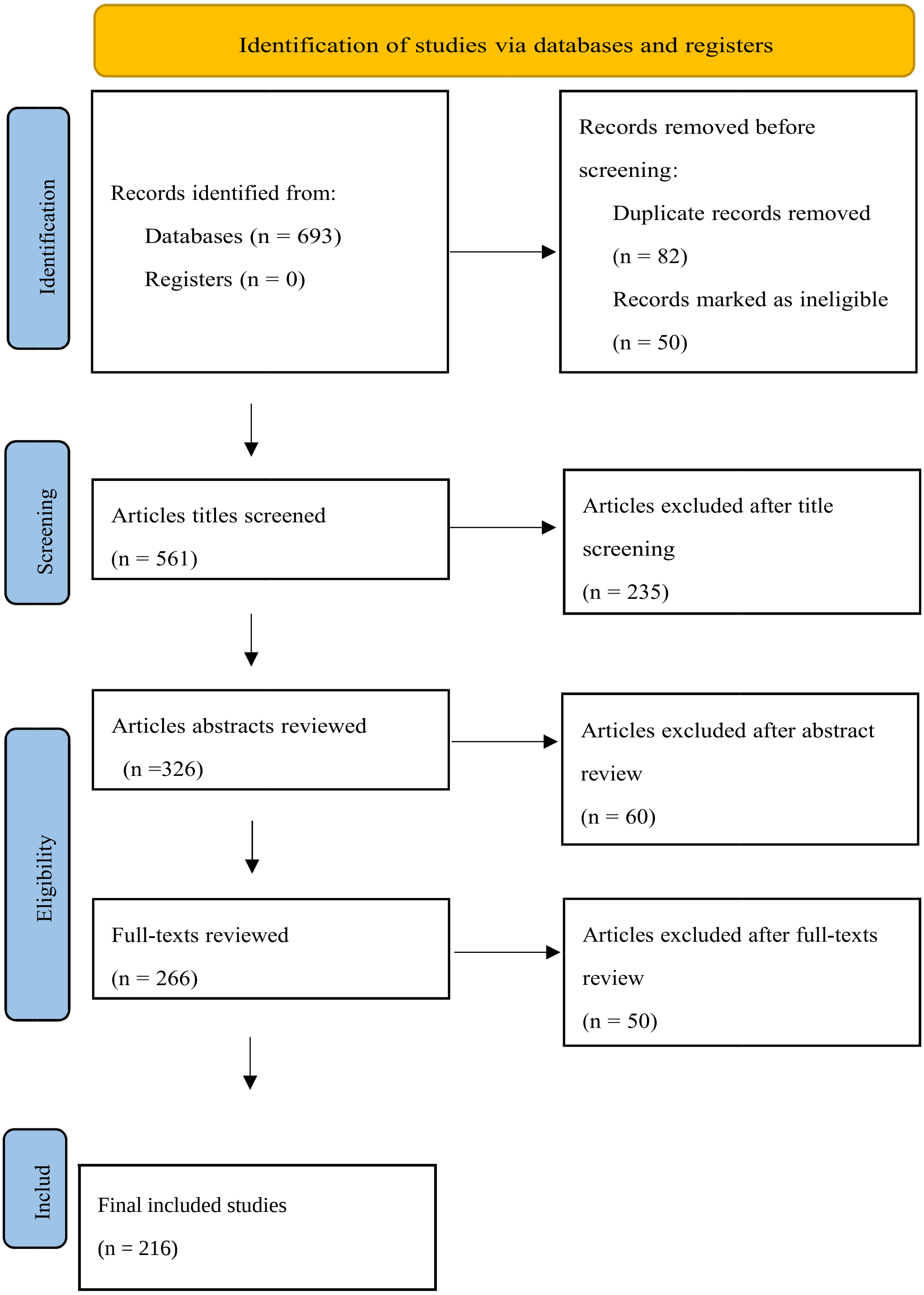

Fig. S1 PRISMA flowchart of the study selection process. The literature was conducted in accordance with the preferred reporting items for systematic reviews and meta-analysis (PRISMA) statement
